# The Bromodomain Inhibitor *N*-Methyl pyrrolidone Prevents Osteoporosis and BMP-Triggered Sclerostin Expression in Osteocytes

**DOI:** 10.3390/ijms19113332

**Published:** 2018-10-25

**Authors:** Barbara Siegenthaler, Chafik Ghayor, Bebeka Gjoksi-Cosandey, Nisarat Ruangsawasdi, Franz E. Weber

**Affiliations:** 1 Center of Dental Medicine, Oral Biotechnology & Bioengineering, University of Zurich, Plattenstrasse 11, 8032 Zürich, Switzerland; barbara.m.siegenthaler@gmail.com (B.S); chafik.ghayor@usz.ch (C.G.); bebeka.cosandey@gmail.com (B.G.-C.); 2 Department of Pharmacology, Faculty of Dentistry, Mahidol University, Bangkok 10400, Thailand; nisarat_mac@hotmail.com

**Keywords:** osteocyte, osteoporosis, sclerostin, bromodomain, *N*-methyl pyrrolidone

## Abstract

(1) Background: In an adult skeleton, bone is constantly renewed in a cycle of bone resorption, followed by bone formation. This coupling process, called bone remodeling, adjusts the quality and quantity of bone to the local needs. It is generally accepted that osteoporosis develops when bone resorption surpasses bone formation. Osteoclasts and osteoblasts, bone resorbing and bone forming cells respectively, are the major target in osteoporosis treatment. Inside bone and forming a complex network, the third and most abundant cells, the osteocytes, have long remained a mystery. Osteocytes are responsible for mechano-sensation and -transduction. Increased expression of the osteocyte-derived bone inhibitor sclerostin has been linked to estrogen deficiency-induced osteoporosis and is therefore a promising target for osteoporosis management. (2) Methods: Recently we showed in vitro and in vivo that NMP (*N*-Methyl-2-pyrrolidone) is a bioactive drug enhancing the BMP-2 (Bone Morphogenetic Protein 2) induced effect on bone formation while blocking bone resorption. Here we tested the effect of NMP on the expression of osteocyte-derived sclerostin. (3) Results: We found that NMP significantly decreased sclerostin mRNA and protein levels. In an animal model of osteoporosis, NMP prevented the estrogen deficiency-induced increased expression of sclerostin. (4) Conclusions: These results support the potential of NMP as a novel therapeutic compound for osteoporosis management, since it preserves bone by a direct interference with osteoblasts and osteoclasts and an indirect one via a decrease in sclerostin expression by osteocytes.

## 1. Introduction

Osteoporosis is a common disease characterized by the deterioration of bone tissue and hence decreasing bone mass. This results in increased bone fragility and higher fracture risk, particularly at the hip, spine and wrist [1]. Hip fractures are the most severe since 80% of these fractures are linked to osteoporosis. Furthermore, hip fractures result in a mortality rate of 20% in the first year and disabilities in 50% of the survivors [2]. The course of osteoporosis is a process of gradual bone loss occurring without symptoms. Today, over 200 million people worldwide are estimated to suffer from this disease. Approximately 30% of all post-menopausal women in Europe and the United States have osteoporosis while at least 40% of these women and 15–30% of all men will experience fragility fractures during their lifetime [3]. Currently available treatments include bisphosphonates, selective estrogen receptor modulators (SERMs), parathyroid hormone (PTH) [4], Denosumab (monoclonal RANKL (Receptor Activator of Nuclear factor Kappa-B Ligand) antibody), Strontium ranelate and supplementation of estrogens, calcium and vitamin D. Even though osteoporosis causes an increased osteoclastic bone resorption activity surpassing the rate of osteoblastic bone formation, most of the above-mentioned treatments are targeting only one of the involved cell type. While there is a diverse armamentarium to serve as inhibitors of the osteoclastic activity, PTH was the first anabolic agent approved by the FDA (Food and Drug Administration (USA)) [5] for the treatment of osteoporosis in 2002. Recently, the FDA approved another PTH analogue (abaloparatide) however, the need for other anabolic agents is still unmet [6]. Strontium ranelate is the single drug currently available targeting both the catabolic and the anabolic side of osteoporosis [7,8]. Unfortunately, all of these treatments come with the cost of undesired side effects making the search for more specific and more potent drugs very urgent.

Recently we showed that *N*-methyl pyrrolidone, a solvent in FDA approved drugs, interferes with the differentiation and activity of osteoblasts and osteoclasts [9,10]. Furthermore, we showed that *N*-methyl pyrrolidone (NMP) acts as a bromodomain inhibitor and plays a role as an epigenetic regulator [11]. Bromodomains are small protein domains recognizing acetylated proteins such as histones. Their binding plays an important role in the remodeling of chromatin structure regulating transcriptional activity [12] and the scaffolding of transcription complexes [13]. Inhibition of bromodomains has already been discussed as promising therapeutic for multiple diseases [14]. The methyl group in NMP combined with the double bonded oxygen atom mimic acetyl-lysine, therefore acting as a bromodomain inhibitor [15,16]. Binding interference studies of the different bromodomain family members by NMP confirmed 70–80% reduced binding capacity of BRD2 (Bromodomain-containing protein 2) and BRD4 [11]. While high affinity bromodomain inhibitors like JQ1 are applied in the micro-molar range, NMP was used in the milli-molar range and can therefore be considered as a low affinity bromodomain inhibitor with additional low affinity binding capability to so far unknown proteins or protein domains. The low affinity of NMP makes this molecule more advantageous than JQ1 because even if both of them inhibit osteoclast differentiation, only NMP is able to enhance the BMP-2-induced osteoblast differentiation [11]. Ultimately, NMP favorably influences the bone formation/bone resorption balance [9,10].

Besides osteoblasts and osteoclasts, osteocytes are the third but most abundant bone cell type in bone and compose up to 95% of all cells in bone tissue [17]. They are differentiating from the mineral-depositing osteoblasts and become embedded within the newly formed osteoid [18,19]. Differentiation of osteoblasts towards osteocytes is indicated by a dramatic morphological change from the polygonal osteoblastic phenotype to a stellate osteocyte extending numerous cellular protrusions to form an interconnected dendritic network [20,21,22]. From the osteocytes located in their lacuna, the dendritic network spreads out within the so called lacuna-canalicular system, is essential for sensing mechanical stimuli [23] and orchestration of the activity of osteoblast and osteoclasts via changes in the molecular signaling through this network [24,25]. Upon mechanical stresses, nitric oxide (NO) is produced and stimulates the COX2 (Cyclooxygenase 2) driven production of Prostaglandin (PGE2) [26,27]. Via further downstream pathways involving estrogen receptors and periostin, the expression of sclerostin is reduced [28,29]. Sclerostin—mainly produced by osteocytes—is encoded by the *SOST* (Sclerosteosis) gene on chromosome 17 in humans [30], is a negative regulator of bone formation and therefore an important regulator of bone mass [31]. It binds to the LRP5/6 receptor and Frizzled co-receptor on the osteoblast cell surface, thereby interfering with Wnt ligand binding and hence blocking osteoblast differentiation and activity [32]. Decreasing osteoblast activity while maintaining osteoclast function leads to a shift of the bone remodeling balance towards bone resorption and bone loss [33]. Extended periods of increased bone resorption; e.g., due to decrease of estrogen, cause severe loss of bone strength with increasing fracture risk [34,35,36]. Targeting sclerostin expression could therefore be a valuable tool for the prevention of osteoporosis and anti-sclerostin antibodies are already investigated [37,38,39]. Moreover, it has been shown previously that bone morphogenetic proteins (BMPs) induce the expression of sclerostin—potentially through the Wnt signaling pathway [40,41]. Increased sclerostin expression under bone anabolic conditions can therefore be considered a negative feedback loop whereby the administered BMPs induce bone formation at osteoblastic levels, and with the parallel induction of sclerostin expression preventing over-stimulation of the anabolic processes or ectopic bone formation.

Following up on our findings showing that NMP inhibits osteoclast differentiation [9], improves osteoblast differentiation and increases bone regeneration [10], the present study aimed to evaluate the effect of NMP on osteocytes.

## 2. Results

### 2.1. NMP Has No Toxic Effect on Osteocyte Like Cells

**Figure 1:**
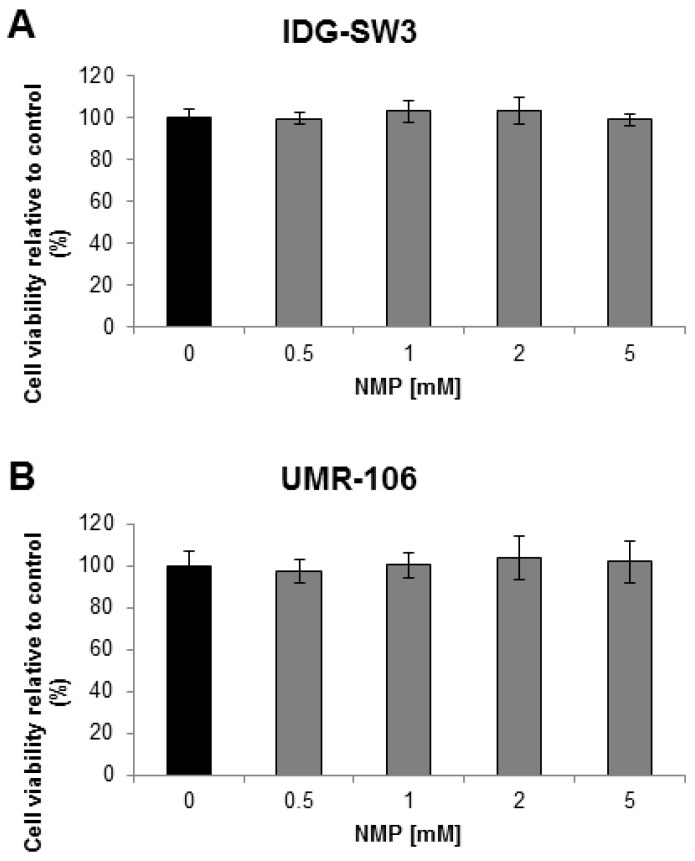
Cell viability dependent on the *N*-methyl pyrrolidone (NMP) concentration. (**A**) Differentiating IDG-SW3 cells were treated with NMP in a range from 0.5–5 mM over a time period of 9 days. (**B**) UMR-106 cells were treated with NMP in a range from 0.5–5 mM over 48 h. WST-1 cell viability assessment at collection time revealed no toxicity at NMP concentrations up to 5 mM.

The response of osteocytes to NMP was evaluated in the mouse immortalized osteocyte cell line IDG-SW3 and the rat osteosarcoma cell line UMR-106. IDG-SW3 cells are a clonal cell line from long-bone chips of mice carrying a Dmp1 promoter driving GFP (Green Fluorescent Protein) crossed with the Immortomouse, which expresses a thermolabile SV40 large T antigen regulated by interferon γ (IFN-γ) [42]. UMR-106 cells are a cloned derivative of a transplantable rat osteogenic sarcoma. Both cell lines are known to express SOST. Therefore, cells from both lines were stimulated with increasing concentrations of NMP and cell viability was assessed using the WST-1 assay. The tested chemical concentrations in the range between 0.5 to 5 mM in differentiating IDG-SW3 cells (Figure 1A) and in UMR-106 cells (Figure 1B) did not reduce cell viability.

### 2.2. NMP Decreases Sclerostin Expression in Osteocyte Like Cells

**Figure 2:**
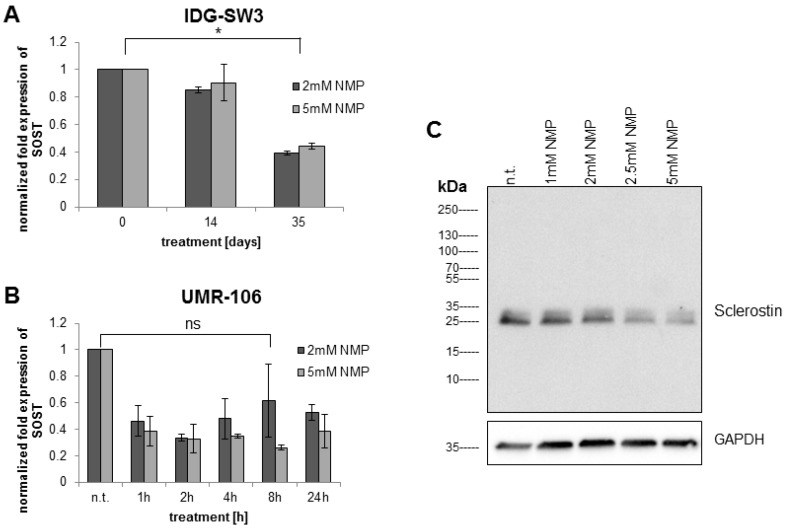
NMP decreases sclerostin mRNA and protein expression. (**A**) Immortalized mouse IDG-SW3 cells were stimulated with 2 mM (dark bars) and 5 mM (light bars) NMP during the whole period of differentiation. (**B**) Rat osteosarcoma UMR-106 cells were incubated with 2 mM (dark bars) and 5 mM (light bars) NMP over a time period of 24 h. (**C**) UMR-106 cells were treated for 8 h with the indicated NMP concentrations in normal culture medium. Whole cell lysates were subjected to Western Blot analysis indicating an NMP concentration-dependent reduction in sclerostin expression. The star indicates statistical significance at α = 0.05, while “ns” indicates non-significance at the same threshold.

To study the potential of NMP to increase bone formation at the level of osteocytic signaling, we investigated the gene expression pattern of *SOST* under NMP-treatment condition. Quantitative reverse-transcription real-time PCR (q RT-PCR) of treated osteocyte-like cells revealed a significant reduction in sclerostin mRNA. In the early stages of osteocyte differentiation (14-day IDG-SW3 cells), NMP modulated only slightly the expression of sclerostin mRNA (Figure 2A). In contrast, when the NMP treatment extends up to 35 days (late osteocyte differentiation), the expression of sclerostin is significantly reduced (*p* < 0.05). Similarly, UMR-106 cells treated with 2 or 5 mM NMP (Figure 2B) displayed a significant reduction in SOST mRNA expression already after 1 h of treatment (non-significant only for 2 mM NMP at 8 h). This reduction stayed constant up to 24 h of stimulation.

The sclerostin reducing effect of NMP was further tested in UMR-106 cells in a titration of chemical up to the initially tested highest concentration of 5 mM. While 1 mM of NMP only slightly reduced sclerostin protein expression, increasing concentrations of NMP induced a concentration-dependent reduction in sclerostin protein expression as measured by Western Blot (Figure 2C).

### 2.3. NMP Prevents BMP-2 Induced Increase in Sclerostin Expression

**Figure 3:**
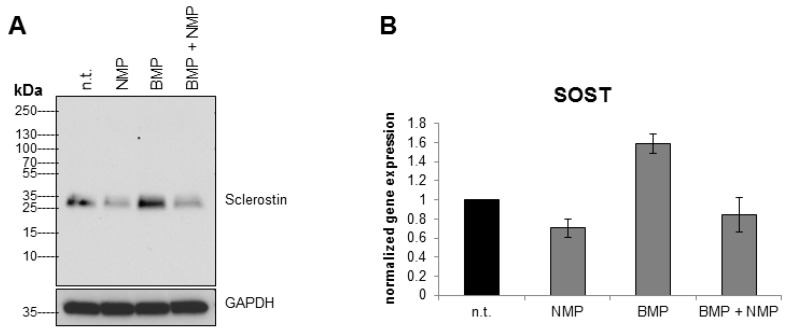
NMP prevents the BMP-2 induced increase in sclerostin expression. Stimulation of UMR-106 cells with 500 ng/mL bone morphogenetic protein 2 (BMP-2) increases sclerostin protein at 8 h (**A**) whereas a combination of BMP-2 and 2 mM NMP decreases sclerostin protein. Illustration of qRT-PCR of the same treatment is presented in (**B**).

It has been reported that BMP-2 treatment of human osteoblastic cells induces sclerostin expression [41]. Here we were interested to test if NMP has the potential to re-balance sclerostin expression after BMP-2 stimulation. Indeed, while BMP-2 stimulation of UMR-106 cells led to an increased sclerostin expression, this response was prevented by a combined stimulation with BMP-2 and NMP (Figure 3A). Figure 3B shows the corresponding SOST mRNA levels determined by q RT-PCR.

### 2.4. Increased Sclerostin Expression in Osteoporosis Induced Animals Can Be Prevented by NMP

**Figure 4:**
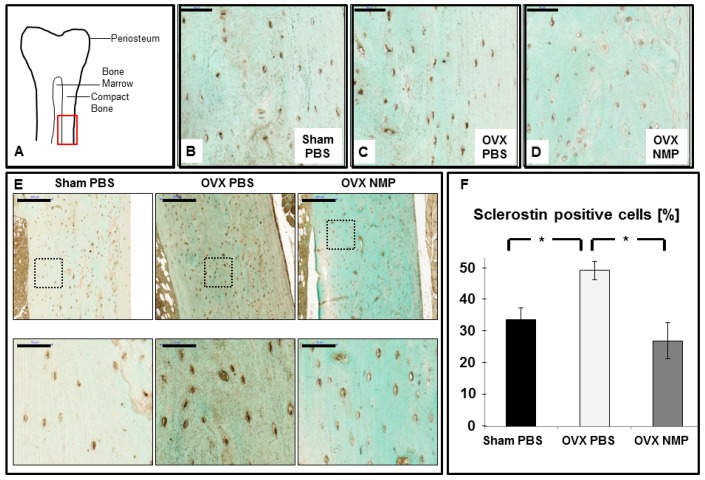
NMP prevents the osteoporosis induced increase in sclerostin expression. Female rats were subjected to ovariectomy (OVX) or control surgery (Sham) and treated with vehicle (PBS) or NMP and femurs were collected for histology. (**A**) Location of image acquisition of histological sections is marked by the red square. (**B**) Sham PBS animals with sclerostin producing osteocytes (brown color). (**C**) OVX animals injected with the vehicle control (PBS). (**D**) OVX animals injected with the chemical NMP. Scale bars in B-D represent 50 μm. (**E**) High and low-magnifications of histological sections stained for sclerostin expression for all three groups as indicated: Sham PBS, OVX PBS and OVX NMP. Scale in the upper panel is 200 µm and in the lower panel 50 µm. The area of the histology of the lower panel is indicated in the respective upper panel by the dashed black rectangle. (**F**) Percentage of sclerostin positive cells. Stars indicate statistical significance at α = 0.05.

Multiple recent studies have shown a relationship between post-menopausal estrogen deficiency and sclerostin levels in humans. To induce osteoporosis in healthy female rats, excision of their ovaries was performed (OVX), while control animals underwent a sham operation (Sham). Paraffin embedded femur sections were immunohistochemically stained for sclerostin protein and the compact area of the bone (red square in Figure 4A) was assessed for sclerostin positive osteocytes (Figure 4A). 

Osteoporosis induced control animals (Figure 4C, OVX PBS) displayed a more numerous and intense sclerostin staining (*p* = 0.018)) compared to the Sham operated, healthy animals (Figure 4B). Furthermore, there was a slight reduction in the number of cells stained positive for sclerostin between OVX animals treated with NMP (Figure 4D, *p* = 0.024) and the Sham control animals (Figure 4B). An additional set of histological section is provided in Figure 4E. Moreover, NMP treatment was able to significantly reduce the number of sclerostin positive cells compared to non-treated OVX animals (*p* = 0.024) (Figure 4F). In control animals (Sham PBS, black bar) about 30% of all osteocytes are sclerostin positive. For osteoporosis induced animals (OVX PBS, white bar) this number increases to 50%. NMP treated animals (OVX NMP, grey bar) contain slightly less (about 25%) sclerostin positive cells than the control.

### 2.5. Bromodomain Inhibition Decreases Sclerostin Expression

**Figure 5:**
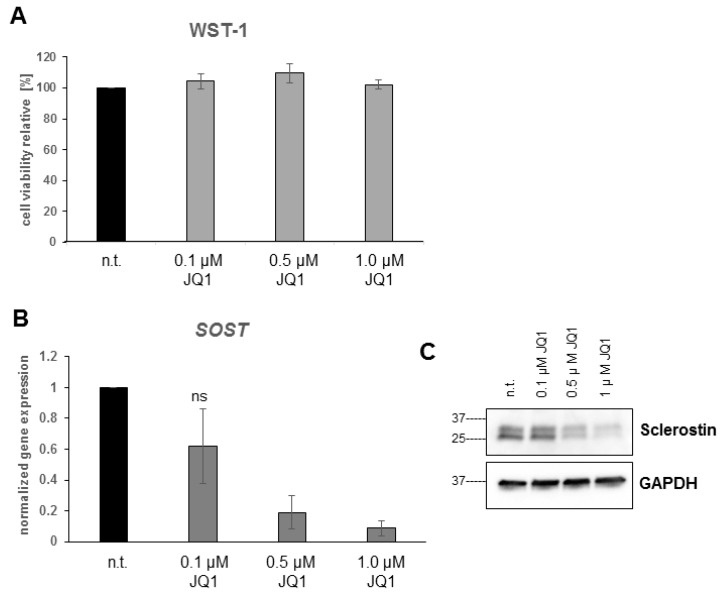
The high affinity bromodomain inhibitor JQ1 decreases sclerostin expression. UMR-106 cells treated for 8 h with a titration of the bromodomain inhibitor JQ1 do not change their metabolic activity (**A**). JQ1 treatment leads to a concentration dependent decrease in SOST mRNA (**B**) and Sclerostin protein (**C**) expression. Error bars indicate standard deviation from three independent experiments. “ns” indicates non-significance at α = 0.05.

The effect of NMP on the sclerostin expression profile was investigated in osteocyte-like UMR-106 cells. The result presented in Figure 2 showed that NMP significantly decrease sclerostin expression within 8 h of stimulation. In order to show that the effect of NMP is due to its bromodomain inhibitor activity, we have treated UMR-106 cells with JQ1, a potent and highly selective bromodomain inhibitor [43]. The mitochondrial activity, which reflect cell viability/toxicity, was evaluated by the WST-1 assay. Up to one μM, JQ1 treatment showed no difference in cell viability (Figure 5A) while the sclerostin gene expression analysis revealed a concentration-dependent decrease (Figure 5B). The results obtained at mRNA level was also confirmed at the protein level by western-blot (Figure 5C). Taken together, these results indicate that the inhibition of bromodomain activity, whether by NMP or by JQ1, is responsible for the negative regulation of sclerostin expression.

## 3. Discussion

Recently we identified the small chemical *N*-methyl pyrrolidone (NMP) as a low-affinity bromodomain inhibitor and potent agent stimulating osteoblastic bone formation [10], while blocking osteoclastic bone resorption [9]. Here we investigated the effect of NMP on gene expression from the main bone cell type, the osteocytes. We focused on sclerostin expression, since various studies have linked osteocytic sclerostin expression to the regulation of bone mass in response to mechanical loading and un-loading of the bone [44]. Sclerostin is expressed in un-loading situations to interfere with the Wnt signaling pathway and thereby blocking the differentiation and activity of osteoblasts. In the present study, stimulation with the low-affinity bromodomain inhibitor NMP to the concentrations tested did not induce toxic responses as measured by the WST-1 assay. In contrast, earlier studies using the known high-affinity bromodomain inhibitor JQ1 demonstrated interference with cell viability and gene transcription [45,46,47]. In our osteocyte-like cell lines we could show that NMP was able to reduce sclerostin expression at the mRNA and protein level in a concentration dependent manner.

Treatment strategies in osteoporotic patients include the administration of bone morphogenetic protein 2 (BMP-2) to stimulate osteoblastic bone formation. These high BMP-2 doses stimulate sclerostin expression in a negative feedback loop to prevent bone overgrowth and ectopic bone formation [40,48]. In our study, BMP-2 administration in UMR-106 cells stimulated the expression of sclerostin, but this increase was suppressed by the co-administration of NMP. This new finding indicates that a co-administration of NMP with BMP-2 would be beneficial, because less of the expensive BMP-2 is required to reach the same bone anabolic effect. Since BMP-2 application is associated with mild to severe side effects, the partial substitution of BMP-2 with NMP might lower the risk for undesired side effects of BMP-2.

Furthermore, besides BMPs increasing sclerostin levels, it has been shown that sclerostin serum levels in postmenopausal women are inversely correlated to the serum estradiol levels [34,35]. A constant estrogen supplementation has been found to reduce sclerostin levels [36,49]. Even though these are clear indications for estrogen regulating the sclerostin levels, the exact mechanisms involved are not resolved yet. To explore the relationship between estrogen deficiency-induced osteoporosis and chemical treatment, an ovariectomized (OVX) rat animal model was employed. Immunohistochemistry of femur sections confirmed an increased sclerostin expression linked to estrogen deficiency in OVX animals, whereas this increase in sclerostin expression was prevented by the systemic injection of NMP. This is of special interest since previous studies have investigated the link between sclerostin expression and bone resorption/formation. While the upregulation of sclerostin expression from osteocytes in disuse conditions was directly linked to bone loss, there was no such correlation observed for the down-regulation of sclerostin upon increased strain [50,51]. Our findings indicate a vital role of the prevention of an increased sclerostin expression in order to maintain bone parameters. These observations further strengthen the role of the low-affinity bromodomain inhibitor NMP as potential new drugs in osteoporosis treatment.

Currently the exact mechanism for NMP’s potential to reduce sclerostin expression and to prevent the BMP-2 or OVX related increase in sclerostin is not clear yet. We hypothesize that the acetyl-lysine mimicking, low-affinity bromodomain inhibitor NMP [11] interferes with the acetyl-recognition by a so far unknown protein involved in sclerostin expression, thereby decreasing its expression. Strong indications for a bromodomain-dependent regulation of the SOST gene is provided by our observation that the established high-affinity bromodomain inhibitor JQ1 induced a concentration dependent decrease in sclerostin expression in UMR-106 cells at 10.000 times lower concentrations than needed with NMP (Figure 5). For both chemicals, the concentrations used are in line with their concentrations needed to inhibit acetyl-lysine binding to bromodomains [11,15,43]. For the high affinity bromodomain inhibitor JQ1, the molecular mechanism has been established [43]. Low affinity inhibitors like NMP, however, have to be applied at concentrations where interference with diverse proteins and signaling pathways can occur. That might have been the reason why JQ1 in contrast to NMP inhibits bone formation by its much higher affinity to bromodomains [11]. The low affinity to bromodomains by NMP, however, can be overwritten by additional effects of NMP in osteoblasts, like the enhancement of the kinase activity for Smads and p38, which sums up to an enhancement of the BMP-signaling in vitro and of bone regeneration in vivo [10]. Therefore, it will be very difficult, if not impossible to dissect all the molecular mechanisms underlying the regulation of sclerostin expression under NMP treatment. 

Various studies have already investigated the regulation of the SOST gene at the level of micro RNAs (Hassan et al. 2012), the upstream proximal SOST promotor [52] and the downstream enhancer element [53]. While in early development, the proximal promotor is strongly controlled via regulation by BMP, Runx2 and Osx predominantly involving DNA methylation [54], the SOST promotor is differentially methylated in mature osteoblasts and mature osteocytes. During the osteoblast-osteocyte transition, the extensive methylations preventing sclerostin expression from osteoblasts is removed, permitting sclerostin expression in osteocytes [17,48,55]. Regulation of the distal enhancer element in the evolutionarily conserved region 5 (ECR5) has recently started to be investigated by multiple groups. They found competitive binding to ECR5 by histone deacetylase 5 (HDAC5) and the myocyte enhancer factor 2C (MEF2C) to influence the acetylation state of lysine 27 on histone 3 (H3K27Ac) and thereby alter the level of sclerostin expression [56,57,58,59,60]. One could speculate that NMP with affinity to the acetyl-binding bromodomain can interfere and inhibit sclerostin expression at this level.

In essence, exposure of osteocytes to NMP is able to reduce sclerostin expression in vitro and in vivo and suggest an anti-osteoporotic effect of the low affinity bromodomain inhibitor NMP mediated on the level of osteocytes by reduced sclerostin expression. Even though we found the established bromodomain inhibitor JQ1 to reduce sclerostin expression in a concentration dependent manner as well, suggesting a bromodomain-dependent inhibition of sclerostin expression, more experiments are needed to identify the exact mechanism underlying the inhibiting effect of bromodomain inhibitors on sclerostin expression.

## 4. Materials and Methods

### 4.1. Cell Culture: Cell Lines

The IDG-SW3 mouse cell line was a kind gift from Prof. Lynda Bonewald (Kansas, MO, USA). IDG-SW3 cells were expanded in proliferation condition at 33 °C in α-MEM (Invitrogen: 22571-020,Carlsbad, CA, USA)) containing 10% heat-inactivated FBS, 100 U/mL of penicillin G, 100 mg/mL of streptomycin and 2500 U/mL of Interferon-γ (Life Technologies, #PMC4031, Lot 989517A, Carlsbad, CA, USA) [42]. To induce IDG-SW3 towards octeocytes, the cells were plated in osteogenic condition at 37 °C with the supplementation of 50 µg/mL of ascorbic acid and 4 mM β-glycerophosphate in the absence of Interferon-γ. Collagen-coated plates (0.15 mg/mL (BD #354236, Franklin Lakes, NJ, USA) in 0.02 M acetic acid) were necessary for both proliferation and differentiation condition. UMR-106 cells (ATCC; Manassas, VA, USA) were cultured in α-MEM medium supplemented with 10% of heat-inactivated FBS, 100 U/mL of penicillin, 50 U/mL of streptomycin at 37 °C.

### 4.2. Cell Viability Assay

Cell viability was assayed using the WST-1 reagent for colorimetric quantification of cellular proliferation, viability and cytotoxicity according to manufacturer’s instruction (Roche Diagnostics, Risch-Rotkreuz, Switzerland). In brief: cells were seeded in 96 well plates and incubated for 24 h, followed by stimulation with NMP (#328634, Sigma-Aldrich, Steinheim, Germany) or JQ1 (#27400, BPS Bioscience, San Diego, IL, USA) as indicated in the figures. WST-1 substrate (1/10th of the total volume = 10 μL) was added to each well and cells were incubated for 2 h. Colorimetric change was measured at 450 nm with 630 nm as a reference.

### 4.3. Quantitative Real-Time RT-PCR

Total RNA was isolated using the RNeasy mini kit (Qiagen, Hilden, Germany) and mRNA was reverse-transcribed into cDNA using the iScript Reverse Transcrition supermix for RT-qPCR (Bio-Rad, Hercules, CA, USA). The resulting cDNA was then used for real-time PCR using the iQ SYBR Green supermix and the myiQ iCycler (both from Bio-Rad). Extension temperature for all primers used was 60 °C. The mouse specific primer pairs to assess GAPDH (Glyceraldehyde-3-phosphate dehydrogenase) and *SOST* in samples from the cell line: IDG-SW3 were purchased from Microsynth (Balgach, Switzerland) (GAPDH forward: 5′-AGGTCGGTGTGAACGGATTTG-3′, GAPDH reverse: 5′-TGTAGACCATGTAGTTGAGGTCA-3′; amplicon length 122 bp; SOST forward: 5′-AGCCTTCAGGAATGATGCCAC-3′, SOST reverse: 5′-CTTTGGCGTCATAGGGATGGT-3′, amplicon length 123 bp). The rat specific primers (QT00199633 for GAPDH, amplicon length 149 bp and QT00418558 for SOST, amplicon length 108 bp) to assess UMR-106 mRNA were purchased from Qiagen.

### 4.4. Western Blot

At collection time, cells were washed with ice cold PBS and frozen at −80 °C until further analysis. Cells were then lysed for 15 min on ice in lysis buffer (Promega, Madison, WI, USA) supplemented with a cocktail of protease and phosphatase inhibitor (Fisher Scientific, Waltham, MA, USA). Collected cell lysate was centrifuged at 14,000× *g* for 15 min and supernatants were transferred to new Eppendorf tubes. Protein concentration was measured using the BCA (BiCinchoninic acid Assay) assay (Thermo Fisher, Waltham, MA, USA). Equal amounts of proteins were run on a 4–20% precast polyacrylamide gel and transferred to a PVDF (Polyvinylidene difluoride) membrane using the precast Trans-Blot turbo stack (both Bio-Rad). Proteins were detected by anti-sclerostin antibody (1:500, R&D AF1589, Minneapolis, MN, USA) and anti GAPDH antibody as loading control (1:1000, Cell signaling D16H11, Danvers, MA, USA), followed by appropriate secondary antibodies coupled to horseradish peroxidase (HRP). After incubation with the electrochemiluminescence (ECL) substrate, signal was detected using a ChemiDoc imaging system (Bio-Rad).

### 4.5. Osteoporosis Rat Model

Healthy female Sprague–Dawley rats were obtained from Charles River laboratories. The rats were adapted to laboratory environment for 2 weeks before the experiment. A total of 30 animals were used for either bilateral laparotomy (Sham, *n* = 10) or bilateral ovariectomy (OVX, *n* = 20). One week after recovering from surgery, the OVX rats were divided into 2 groups: OVX receiving vehicle (OVX Veh, *n* = 10) and OVX receiving NMP (OVX NMP, *n* = 10). Treatment via intraperitoneal injection was started 1 week after OVX and lasted for 15 weeks. The administered dose was weekly adjusted to animal body weight. Femurs were collected immediately following sacrifice and adherent soft tissue was removed. Bone samples were fixed, decalcified (10% EDTA at 37 °C) and embedded in paraffin (Sophistolab AG, Muttenz, Switzerland) for further analysis. All animal procedures met the ARRIVE guidelines were approved by the Animal Ethics Committee of the local authorities (Veterinäramt, Canton Zurich, project codes: 40/2012 (approved on the 2 April 2012) and 068/2015 (approved on the 31 July 2015), and follow the EU Directive 2010/63/EU for animal experiments.

### 4.6. Immunohistochemistry (IHC)

Paraffin sections from long bones were de-paraffinized and rehydrated in a series of decreasing percentage of Ethanol. After permeabilization with 0.1% NP-40 in PBS for 10 min, sections were blocked in TBS supplemented with 10% normal serum and 1% BSA for 2 h at room temperature in a humidified chamber. Anti sclerostin antibody (Abcam ab63097, 1:25, Cambridge, UK) was added directly onto the sections in TBS containing 1% BSA and incubated at 4 °C overnight (negative controls did not contain primary antibody). After washing off the primary antibody in TBS + 0.025% Triton, peroxide background signal is reduced by an incubation in 0.3% H_2_O_2_ in TBS for 15 min. HRP conjugated secondary antibodies are incubated for 1 h at room temperature in TBS + 1% BSA (Abcam ab97085, 1:200) and signal is detected with 3,3′-diaminobenzidine tetrahydrochlordie (DAB) for 5–10 min. Slides were counterstained using light green reagent, dehydrated in a series of increasing ethanol concentration, fixed in Xylol and mounted with Eukitt. Images were captured using an automated slide scanner form the Center of light microscopy at the University of Zürich (ZMB). Signal quantification was performed in a blinded manner.

### 4.7. Statistical Analysis

Experiments were performed in at least three independent experiments. Results are expressed as mean ± Standard Deviation (SD). Statistical analysis was performed using the Student’s *t* test and considered significant with *p* < 0.05 (*). 

## Abbreviations

ALP	Alkaline phosphatase
BCA	BiCinchoninic acid Assay
BMP	Bone morphogenetic protein
bp	Base pair
BRD2	Bromodomain-containing protein 2
COX	Cyclooxygenase
FDA	Food and Drug Administration (USA)
GAPDH	Glyceraldehyde-3-phosphate dehydrogenase
GFP	Green fluorescent protein
JQ1	(6*S*)-4-(4-Chlorophenyl)-2,3,9-trimethyl-6H-thieno[3,2-f][1,2,4]triazolo[4,3-a][1,4]diazepine-6-acetic acid 1,1-dimethylethyl ester
LEP	Leptin
NMP	*N*-methyl pyrrolidone
NO	Nitric oxide
Osx	Osterix
OVX	Ovariectomy
PGE	Prostaglandine
PTH	Parathyroid Hormone
PVDF	Polyvinylidene difluoride
RANKL	Receptor Activator of Nuclear factor Kappa-B Ligand
*SOST*	Sclerosteosis gene
WST	(4-[3-(4-Iodophenyl)-2-(4-nitro-phenyl)-2H-5-tetrazolio]-1,3-benzene disulfonate)
